# Chromosome Damage in Relation to Recent Radiation Exposure and Radiation Quality in Nuclear Power Plant Workers

**DOI:** 10.3390/toxics10020094

**Published:** 2022-02-18

**Authors:** Yang Jee Kim, Joong Won Lee, Yoon Hee Cho, Young Joo Choi, Younghyun Lee, Hai Won Chung

**Affiliations:** 1 Da Vinci College of General Education, Chung-Ang University, 84 Heukseok-ro, Dongjak-gu, Seoul 06974, Korea; 2 Department of Research and Planning, Korea National Institute of Health, Chungju 28159, Korea; lasthitter@gmail.com; 3 Department of Biomedical and Pharmaceutical Sciences, The University of Montana, Missoula, MT 59812, USA; unicho3@gmail.com; 4 School of Public Health Seoul National University, 1 Gwanak-ro, Gwanak-gu, Seoul 08826, Korea; yjchoi1230@daum.net (Y.J.C.); younghyun.lee.0123@gmail.com (Y.L.); chunghw@snu.ac.kr (H.W.C.)

**Keywords:** ionizing radiation, nuclear power plant, chromosome aberration, multi-aberrant cells, chronic neutron exposure

## Abstract

Ionizing radiation is a well-known carcinogen that causes genomic instability. However, the biological and carcinogenetic effects of occupational radiation exposure at low doses have not been extensively studied. The aim of this study was to assess chromosomal instability in power plant workers exposed to occupational radiation at low doses in South Korea. Chromosomal aberrations in the lymphocytes of 201 nuclear power plant workers and 59 sex-matched controls were measured. Chromosomal aberrations in the lymphocytes of 201 nuclear power plant workers (mean age: 41.4 ± 10.0 years) and 59 sex-matched controls (mean age: 47.2 ± 6.0 years) were measured. A total of 500 metaphases for each subject were scored randomly. The means of recent 1.5-year, recent 5.5-year, and cumulative exposed radiation doses among workers were 8.22 ± 7.0 mSv, 30.7 ± 22.0 mSv, and 158.8 ± 86.1 mSv, respectively. The frequency of chromosome-type and chromatid-type aberrations was significantly higher in workers than that in the control group (*p* < 0.001), and the frequency of chromosome-type aberrations among workers increased in a radiation dose-dependent manner (τ = 0.16, *p* = 0.005). Poisson regression analyses revealed that chromosome-type aberrations were significantly associated with recent 1.5-year dose after adjusting for confounding variables such as age, smoking, and alcohol intake, even when only the exposed worker was considered. Frequency of multi-aberrant cells (two or more chromosome aberrations within a cell) increased according to cumulative neutron exposure. Our study demonstrates that chromosome damage can be induced in nuclear power plant workers occupationally exposed to ionizing radiation at low doses below the occupational permissible dose limit. Furthermore, an increase in multi-aberrant cells may provide evidence for chronic neutron exposure in nuclear power plant workers. This study was performed to obtain baseline data for a surveillance program of workers occupationally exposed to ionizing radiation long-term.

## 1. Introduction

It is well known that ionizing radiation (IR) produces DNA damage and chromosomal alterations, indicating induction of genomic instability. Genomic instability is characterized by an increased tendency to alter the genome, subsequently increasing cancer risk [1,2,3,4]. Chromosomal aberrations are a striking form of radiation-induced genomic instability [5]. In particular, dicentric chromosomes, a chromosome-exchange aberration, have been considered a gold standard marker for IR exposure and may play an important role in the early stage of cancer progression [6]. Chromosome aberration analyses in peripheral blood are widely used to estimate the absorbed dose in biological dosimetry and are a reliable biomarker for predicting cancer risk in healthy populations [7,8]. Therefore, monitoring increased chromosome aberrations in the peripheral blood from workers and the public who are occupationally or accidently exposed to IR may be useful to survey workers and the public for their susceptibility to cancer development [9].

Although IR is a well-known carcinogen, the question of biological and carcinogenic effects of IR exposure at low doses has not been fully understood. Therefore, the risk of low-dose IR exposure has been increasingly highlighted as a potential concern regarding environmental, therapeutic, and occupational exposures [10]. In particular, research on low-dose radiation is needed on individuals occupationally exposed to IR to better understand the biological and carcinogenic effects of IR exposure at low doses. Several studies have reported that occupational exposure to low-dose radiation, even at a few hundred mSv, can induce chromosomal damage, including micronuclei, sister chromatid exchange, and chromosome aberrations [11,12,13,14].

Occupational IR exposure has decreased in recent decades and is now far below the regulatory limit of 20 mSv (2 rem) per year, averaged over five years (100 mSv/5 years) [15]. However, exposure to IR may result in cumulative effects with increasing duration of employment in workers occupationally exposed to low-dose IR at nuclear power plants. Nuclear power plant workers are exposed to various types of radiation, such as gamma-ray, tritium, and neutrons. In general, densely ionizing radiation (high-LET radiation), such as α-particles and neutrons, generates multiple damaged sites in DNA, and consequently, multi-aberration could be induced. Although the presence of chromosome damage itself, in workers occupationally exposed to IR, does not necessarily lead to immediate adverse health effects, high levels of chromosome aberrations are thought to indicate an increased risk for cancer. However, most studies on workers exposed to radiation were conducted using total exposure radiation, not considering high LET, especially neutrons. In the present study, 201 workers exposed to low dose IR from four nuclear power plants in South Korea and 59 controls were analyzed using conventional chromosome aberration analysis methods to assess the level of chromosome damage under exposure conditions below the currently accepted level of 20 mSv per year for the workplace.

## 2. Materials and Methods

### 2.1. Study Population

The study population was comprised of 201 male workers occupationally exposed to low doses of IR from four nuclear power plants located in South Korea (Kori, Wolsong, Yonggwang, and Ulchin) and 59 sex-matched volunteers. We recruited nuclear power plant workers exposed to 100 mSv IR as a recorded cumulative dose, during their whole work experience, and collected biospecimens and exposure data from the participants from 2007 to 2009 in this study. In 2010, high-exposure workers who participated in regular inspections were included (4.54 ± 0.46, 10.16 ± 1.21, respectively, in 2007–2009 and 2010). The controls were office workers who had never been occupationally exposed to IR; their blood was collected during the same periods as that of the nuclear power plant workers. Information regarding smoking, drinking habits, medical history, drug intake, and duration of occupational exposure to radiation (years of employment) was obtained via personal interviews. Neither the nuclear power plant workers nor the controls had a personal medical history of cancer or genetic disease. The study participants were not exposed to medical irradiation and had no prescription medications in a month prior to the study. To determine the occupational radiation dose among workers, official personal dosimetry records were obtained from the Korean National Dose Registry, managed by the Korea Radio-Isotope Association (KRIA). The doses resulting from external and internal radiation and internal were combined to determine the total effective dose [16]. In our study population, the external doses account for about 97% of the total exposed doses while internal exposures represented only 3%.

### 2.2. Chromosome Aberration Analysis

Heparinized blood (1 mL) was added to 9 mL RPMI 1640 medium (Gibco BRL, Grand Island, NY, USA) containing 10% fetal bovine serum (Sigma Chemical Co., St. Louis, MO, USA), penicillin (100 U/mL), streptomycin (100 μL/mL), phytohemagglutinin M (1%), and bromodeoxyuridine (BrdU). The cultures were incubated at 37°C for 48 h in an atmosphere containing 5% CO2. Colcemid (Sigma, 0.1 μL/mL) was added to the cultures 3 h before harvest. Chromosome preparations were performed according to the standard procedure [17]. Slides were coded blindly and 500 metaphases for each subject were scored randomly.

Chromosomal aberrations were evaluated based on the following 4 categories: chromatid-type deletions, chromatid-type exchanges, chromosome-type deletions, and chromosome-type exchanges. Gaps were not scored as aberrations. Cells with two or more chromosome-type aberrations were considered multi-aberrant cells. After chromosome aberration analysis, the radiation records were linked to a code number for data analysis.

Fluorescence plus Giemsa (FPG) staining for sister chromatids [18] was also performed to differentiate between metaphases in the first and second cell division, and only first division metaphases were selected. The first metaphase was observed in 87–95% (average 90.5%) of samples using BrdU incorporation.

### 2.3. Statistical Analysis

Statistical analyses were carried out using SAS 8.1 statistical program for Windows (SAS Institute, Inc., Cary, NC). Differences in the frequency of chromosome aberrations between nuclear power plant workers and controls were analyzed by the Mann–Whitney U-test. The association between aberration yield and radiation dose was tested by the Kendall rank correlation coefficient (τ). Correlations of aberration yield with years of employment or recorded doses were tested by the Pearson correlation and p for trend. Poisson regression analysis was applied to evaluate the independent association between the yield of chromosome aberrations and various variables, including age, smoking status, alcohol intake, and radiation doses. A goodness-of-fit test for Poisson assumption and dispersion test for detecting heterogeneity for Poisson distribution were done with chromosomal aberrations. The association between the presence of multi-aberrations and neutron exposure was analyzed by logistic regression. The criterion for significance was set at *p* < 0.05.

## 3. Results

The general characteristics of the study population are listed in Table 1. All subjects in the study were healthy males, and the mean age of the nuclear power plant workers was higher than that of the controls. The age of the workers and controls ranged from 24 to 65 years, with a mean of 41.4 ± 10.0 years, and 29 to 59 years, with a mean of 47.2 ± 6.0 years, respectively. There were no significant differences in patterns of smoking status between workers and controls; however, workers consumed more alcohol than the controls. The mean duration of employment for the exposed workers was 19.9 ± 6.2 years and ranged from 3 to 32 years. Dosimetry records for the duration of employment show that the mean recent 1.5-year, recent 5.5-year, and cumulative exposed radiation doses among workers were 8.22 ± 7.0 mSv (range, 0–33.5), 30.7 ± 22.0 mSv (range, 0–81.7), and 158.8 ± 86.1 mSv (range, 1.98–403.13), respectively. Differences in the frequency of chromosome aberrations between exposed workers and the control group were statistically significant for both chromatid-type (deletion only) and chromosome-type aberrations (*p* < 0.001, Table 2). The mean values of total chromosome aberrations were 8.42/500 metaphase cells in workers and 4.22/500 metaphase cells in controls.

**Figure 1:**
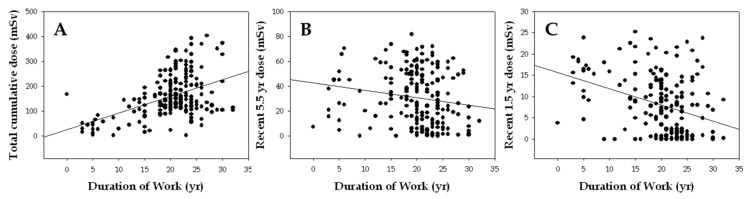
Correlation between years of employment and cumulative dose (**A**), recent 5.5-year dose (**B**) and recent 1.5-year dose (**C**).

Total cumulative radiation dose shows a positive correlation with years of employment (*r* = 0.37, *p* < 0.001), but the recent 5.5-y dose (*r* = −0.20, *p* = 0.009) and recent 1.5-y dose (*r* = −0.23, *p* = 0.003) are inversely correlated (Figure 1).

**Figure 2:**
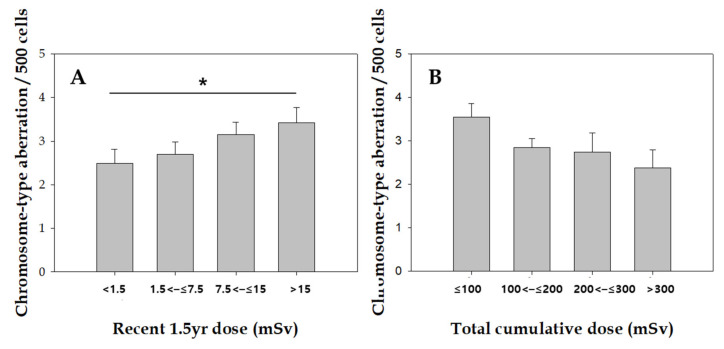
Association of recent 1.5-y dose with chromosome-type aberration. Error bars represent the standard deviation of the mean. *, *p* < 0.05 (*p* for trend).

The frequency of chromosome aberrations by the different radiation doses is shown in Figure 2. The frequency of chromosome-type aberrations was significantly higher for the recent 1.5-y dose (τ = 0.17, p trend = 0.04), whereas no significant relationship between chromosome-type aberrations and recent 5.5-year dose was found (data not shown). There was a borderline significant inverse dose response between cumulative radiation dose and chromosome-type aberrations among workers (τ = −0.13, *p* = 0.06).

As shown in Table 3. when Poisson regression analysis was applied only to the exposed group, the recent 1.5-year dose was significantly associated with frequency of chromosome-type aberration after adjusting for age, smoking status, and alcohol intake (*p* = 0.032).

**Figure 3:**
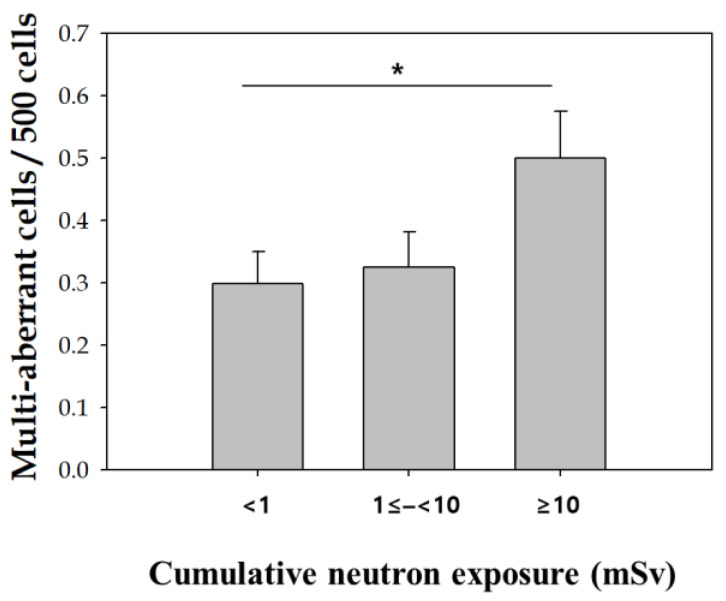
Association of multi-aberrant cells with neutron exposure. Error bars represent the standard deviation of the mean. *, *p* < 0.05 (*p* for trend).

We also examined the distribution of multi-aberrant cells (cells with 2 or more chromosome-type aberrations) in the workers and controls. Multi-aberrant cells appeared more frequently in exposed workers than that in the controls. Fifty-seven exposed workers (28.4%) had multi-aberrant cells, whereas three controls (5.1%) had multi-aberrant cells. Furthermore, the frequency of multi-aberrant cells tended to increase according to cumulative neutron exposure, and there was a significant different in multi-aberrant cell frequency between cumulative neutron exposure doses (< 1 vs. ≥ 10) (Figure 3).

The association between the presence of multi-aberrations and neutron exposure was analyzed by logistic regression (Table 4). Multi-aberrant cells occurred 1.67 times more in people with 10 mSv or more compared to those with total neutron exposure less than 1 mSv, even after adjusting for age, alcohol intake, and smoking.

## 4. Discussion

IR can induce specific types of cancer, particularly leukemia, and chromosome aberrations are frequently found in many cancer types [19]. The potential for chromosome aberrations to be used as a biomarker in cancer is supported by recent epidemiological studies showing a positive association between a high frequency of chromosomal aberrations and increased cancer [1,2,3,4]. The use of chromosome aberrations as biomarkers for accurate radiation dose reconstruction at the individual level is uncertain; however, high levels of chromosome aberrations apparently indicate potential risk. Therefore, chromosome aberrations can likely be used as an effective early marker of radiation exposure at the population level in long-term follow-up studies.

This study was performed to obtain baseline data for a surveillance program of workers occupationally exposed to IR long-term. Although the level of exposure was below the accepted annual limit of 20 mSv in this study, the yields of both chromosome-type and chromatid-type aberrations were significantly higher in the radiation workers than those in the controls. The frequency of chromosome-exchange aberrations (i.e., dicentric chromosome) was 0.94/500 cells in the exposed workers and 0.14/500 cells in control subjects. These results agree with our previous study [20] and several other studies [13,14,21,22]. The levels of dicentric chromosomes in the controls in this study were a little lower than those in other published studies; however, they remained within the range of earlier studies (0.2/1000 cells) [23].

We found no association between age, smoking status, or alcohol intake and the incidence of any type of chromosome aberration, although the age effect on chromosome damage is still controversial [20,24,25]. Furthermore, an inverse relationship was observed between work duration and cumulative dose in our study (Figure 1). This is likely attributable to changing working conditions or job position (i.e., promotion, thus new employees are more likely working in areas where they are exposed to IR), suggesting that duration of work is not a proper surrogate for total individual radiation dose [26].

We analyzed the association between aberration yield and total cumulative, recent 5.5-year, and recent 1.5-year dose to determine whether radiation-exposed time points are important contributors to the frequency of chromosome aberrations in workers occupationally and chronically exposed to IR. Our data show a dose-dependent increase in chromosome-type aberrations in relation to recent 1.5-year dose, but not recent 5.5-y or cumulative radiation doses. Poisson regression analyses revealed a significant association between the frequency of chromosome-type and recent 1.5-year dose among radiation-exposed workers after adjusting for age, smoking status, and alcohol intake. These data suggest that recent radiation exposure more effectively causes chromosomal damage, and chromosome aberrations may serve as biomarkers for recent exposure to IR, but not chronic exposure. Twenty years after the Chernobyl nuclear accident, the frequency of dicentrics in people exposed to radiation decreased to the background level [27]. This can be explained by the level of unstable chromosome aberrations, such as decreased dicentric chromosomes in the peripheral blood lymphocytes with time.

Of particular interest, the frequency of multi-aberrant cells in exposed workers was higher than that of the control group, and the frequency of multi-aberrant cells also increased with cumulative neutron exposure. It Indicates that neutron generated multi-aberrant cells through damaging multiple sites in DNA. The appearance of multi-aberrant cells is expected from high linear energy transfer (LET) radiation [24,28] and useful for classifying radiation type, even though the mechanisms of multi-aberrant cells are not yet fully understood. It is well known that high LET radiation, such as neutrons, more effectively induces complex chromosome aberrations than low LET radiation [29] Moreover, In this study, we considered neutron as the main source of high LET radiation exposure, neutrons induced an increase of multi-aberration. [30,31]. Multi-aberrant cell formation resulting from high LET irradiation in pilots and astronauts has been proposed as a mechanism of toxicity [28,30]. This study has a limitation focusing on only unstable chromosomal aberrations caused by radiation exposure. Fluorescence in situ hybridization (FISH)-based translocation assay is a representative methodology to assess stable chromosomal aberrations [32], which could help to study further the impact of their occupational radiation exposure on stable chromosomal damages. Specifically, the 24-multicolor FISH technique would allow evaluating complex chromosome exchanges induced by high LET radiation and thereby find the contribution of neutron exposure to the chromosomal damages of our subjects [33,34,35] Another limitations to this study includes the low number of multi-aberrant cells, the inexact criteria for multi-aberrant cells, and the fact that insignificant amounts of neutron exposure contribute to all external effective doses.

## 5. Conclusions

In this study, the recent 1.5-year dose was significantly associated with frequency of chromosome-type aberration in nuclear power plant workers, supporting that the applicability of unstable chromosome aberration as biomarkers for monitoring recent radiation exposure. Therefore, chromosomal aberration analysis could be used as a biodosimetry supplementing the physical dosimetry and as a biological indicator that can detect a disease before it occurs to evaluate the human health effects of workers exposed to radiation. In addition, we found an increase in the number of multi-aberrant cells in workers exposed to radiation, suggesting that is possible evidence for chronic neutron exposure in nuclear power plant workers.

## Figures and Tables

**Table 1:** General characteristics of study population.

Variables	No. of Subjects (%)		*p*-Value
	Controls	Workers	
Number	59	201	
Age (mean ± SD, years)	41.4 ± 10.0	47.2 ± 6.0	0.001 ^a^
≤50	47 (79.7)	135 (67.2)	0.07 ^b^
>50	12 (20.3)	66 (32.8)	
Smoking status (mean ± SD, pack-years)	14.6 ± 13.5	12.6 ± 11.3	0.52 ^a^
Never smoker	26 (44.1)	49 (24.4)	0.001 ^b^
Currently smoking	28 (47.5)	78 (38.8)	
Ex-smoker	5 (8.5)	74 (36.8)	
Alcohol intake			
No	22 (37.3)	39 (19.4)	0.01 ^b^
Yes	37 (62.7)	162 (80.6)	
Duration of employment (mean ± SD, years)	-	19.9 ± 6.2	
≤20	-	99 (49.3)	
20–25	-	75 (37.3)	
>25	-	27 (13.4)	
Dosimetry radiation dose			
Recent 1.5-year (mean ± SD, mSv)	-	8.23 ± 7.01	
Recent 5.5-year (mean ± SD, mSv)	-	30.68 ± 22.01	
Cumulative dose (mean ± SD, mSv)	-	158.78 ± 86.05	

SD, standard deviation. ^a^ Determined by Mann–Whitney U-test. ^b^ Determined by *x^2^* test.

**Table 2:** Frequencies of chromosomal aberrations in nuclear power plants workers and controls.

Types of Chromosome Aberration	Controls			Workers			
	Mean/500 Cells	SEM	Range	Mean/500 Cells	SEM	Range	*p*-Value
Number	59			201			
Chromatid-type deletion	3.59	0.32	0–12	5.38	0.19	0–17	<0.001 *
Chromatid-type exchange	0.09	0.04	0–2	0.05	0.02	0–1	0.131
Chromosome-type deletion	0.41	0.10	0–3	2.02	0.14	0–14	<0.001 *
Chromosome-exchange	0.14	0.05	0–2	0.94	0.07	0–6	<0.001 *
Total aberration	4.22	0.35	0–13	8.42	0.26	1–25	<0.001 *

SEM, standard error of the mean. * Significantly different from control subjects (determined by Mann–Whitney U-test, *p* < 0.05).

**Table 3:** Poisson regression analysis for chromosome-type aberrations associated with age, smoking status, alcohol intake, and dose in exposed group.

Outcome	Variable	*β* Coefficient	95% C.L.	*p*-Value
Chromosome-type aberration	Age (in years)	−0.002	−0.060, 0.016	0.722
	Smoking status (0,1) ^1^	0.080	−0.135, 0.294	0.467
	Alcohol intake (0,1) ^2^	−0.018	−0.271, 0.234	0.887
	Total cumulative Dose(mSV)	−0.001	−0.0024, 0.003	0.126
Chromosome-type aberration	Age	−0.002	−0.060, 0.016	0.831
	Smoking (0,1) ^1^	0.090	−0.124, 0.303	0.411
	Alcohol (0,1) ^2^	−0.020	−0.272, 0.231	0.874
	Recent 1.5-y Dose(mSV)	0.008	0.0014, 0.032	0.032

^1^ Smoking status: 0, never smoker;1, smoking. ^2^ alcohol intake: 0, never; 1, current.

**Table 4:** Logistic regression analysis for multi-aberrant cell associated with age, smoking status, alcohol intake, and cumulative neutron exposure.

Outcome	Variable	RR	95% C.L.	*p*-Value
Presence of multi-aberrant cells	Age (in years)	1.00	0.92, 1.03	0.351
	Smoking status (0,1) ^1^	1.00	0.51, 2.00	0.981
	Alcohol intake (0,1) ^2^	1.35	0.58, 3.07	0.503
	Neutron exposure (mSv)			
	<1	reference		
	1≤–<10	1.02	0.53, 1.98	0.947
	≥10	1.67	0.37, 7.48	0.502

RR: Relative risk, ^1^ smoking status: 0, never smoker; 1, smoking. ^2^ alcohol intake: 0, never; 1, current.

## Data Availability

The data presented in this study are available on request from the corresponding author.

## References

[1] Bonassi S., Abbondandolo A., Camurri L., Dal Prá L., De Ferrari M., Degrassi F., Forni A., Lamberti L., Lando C., Padovani P. (1995). Are chromosome aberrations in circulating lymphocytes predictive of future cancer onset in humans? Preliminary results of an Italian cohort study. Cancer Genet. Cytogenet..

[2] Hagmar L., Brøgger A., Hansteen I.-L., Heim S., Högstedt B., Knudsen L., Lambert B., Linnainmaa K., Mitelman F., Nordenson I. (1994). Cancer risk in humans predicted by increased levels of chromosomal aberrations in lymphocytes: Nordic study group on the health risk of chromosome damage. Cancer Res..

[3] Liou S.-H., Lung J.-C., Chen Y.-H., Yang T., Hsieh L.-L., Chen C.-J., Wu T.-N. (1999). Increased chromosome-type chromosome aberration frequencies as biomarkers of cancer risk in a blackfoot endemic area. Cancer Res..

[4] Rossner P., Boffetta P., Ceppi M., Bonassi S., Smerhovsky Z., Landa K., Juzova D., Šrám R.J. (2005). Chromosomal aberrations in lymphocytes of healthy subjects and risk of cancer. Environ. Health Perspect..

[5] Kaplan M.I., Limoli C.L., Morgan W.F. (1997). Perpetuating radiation-induced chromosomal instability. Radiat. Oncol. Investig. Clin. Basic Res..

[6] Sasaki M.S., Miyata H. (1968). Biological dosimetry in atomic bomb survivors. Nature.

[7] Bonassi S., Norppa H., Ceppi M., Strömberg U., Vermeulen R., Znaor A., Cebulska-Wasilewska A., Fabianova E., Fucic A., Gundy S. (2008). Chromosomal aberration frequency in lymphocytes predicts the risk of cancer: Results from a pooled cohort study of 22 358 subjects in 11 countries. Carcinogenesis.

[8] Fucic A., Bonassi S., Gundy S., Lazutka J., Sram R., Ceppi M., Lucas J.N. (2016). Frequency of acentric fragments are associated with cancer risk in subjects exposed to ionizing radiation. Anticancer. Res..

[9] Carrano A., Natarajan A. (1988). International Commission for Protection Against Environmental Mutagens and Carcinogens. ICPEMC publication no. 14. Considerations for population monitoring using cytogenetic techniques. Mutat. Res..

[10] Ma S., Liu X., Jiao B., Yang Y., Liu X. (2010). Low-dose radiation-induced responses: Focusing on epigenetic regulation. Int. J. Radiat. Biol..

[11] Evans H., Buckton K., Hamilton G., Carothers A. (1979). Radiation-induced chromosome aberrations in nuclear-dockyard workers. Nature.

[12] Balakrishnan S., Rao S.B. (1999). Cytogenetic analysis of peripheral blood lymphocytes of occupational workers exposed to low levels of ionising radiation. Mutat. Res./Genet. Toxicol. Environ. Mutagen..

[13] Lindholm C. (2001). Stable and unstable chromosomal aberrations among Finnish nuclear power plant workers. Radiat. Prot. Dosim..

[14] Maffei F., Angelini S., Forti G.C., Lodi V., Violante F.S., Mattioli S., Hrelia P. (2002). Micronuclei frequencies in hospital workers occupationally exposed to low levels of ionizing radiation: Influence of smoking status and other factors. Mutagenesis.

[15] Publ I. (2008). 2007 Recommendations of the International Commission on Radiological Protection. Ann. ICRP.

[16] Lee B.-I., Kim S.-I., Suh D.-H., Jin Y.-W., Kim J.-I., Choi H., Lim Y.-K. (2010). Radiation dose distribution for workers in South Korean nuclear power plants. Radiat. Prot. Dosim..

[17] Manual A. (2001). Cytogenetic analysis for radiation dose assessment. Tech. Rep. Ser.-Int. At. Energy Agency.

[18] Perry P., Wolff S. (1974). New Giemsa method for the differential staining of sister chromatids. Nature.

[19] Erdoğan G., Aksoy M. (1973). Cytogenetic studies in thirteen patients with pancytopenia and leukaemia associated with long-term exposure to benzene. New Istanb. Contrib. Clin. Sci..

[20] Chung H.W., Ryu E.K., Kim Y.J., Ha S.W. (1996). Chromosome aberrations in workers of nuclear-power plants. Mutat. Res./Fundam. Mol. Mech. Mutagen..

[21] Braselmann H., Schmid E., Bauchinger M. (1994). Chromosome aberrations in nuclear power plant workers: The influence of dose accumulation and lymphocyte life-time. Mutat. Res. /Fundam. Mol. Mech. Mutagen..

[22] Baudin C., Bernier M.-O., Klokov D., Andreassi M.G. (2021). Biomarkers of Genotoxicity in Medical Workers Exposed to Low-Dose Ionizing Radiation: Systematic Review and Meta-Analyses. Int. J. Mol. Sci..

[23] Bauchinger M., Kolin-Gerresheim J., Schmid E., Dresp J. (1980). Chromosome analyses of nuclear-power plant workers. Int. J. Radiat. Biol. Relat. Stud. Phys. Chem. Med..

[24] Ramsey M.J., Moore II D.H., Briner J.F., Lee D.A., Olsen L.A., Senft J.R., Tucker J.D. (1995). The effects of age and lifestyle factors on the accumulation of cytogenetic damage as measured by chromosome painting. Mutat. Res./DNAging.

[25] Rozgaj R., Kašuba V., Šimić D. (2002). The frequency of dicentrics and acentrics and the incidence of rogue cells in radiation workers. Mutagen..

[26] Hadjidekova V.B., Bulanova M., Bonassi S., Neri M. (2003). Micronucleus frequency is increased in peripheral blood lymphocytes of nuclear power plant workers. Radiat. Res..

[27] Montoro A., Sebastià N., Candela-Juan C., Barquinero J.F., Soriano J.M., Almonacid M., Alonso O., Guasp M., Marques-Sule E., Cervera J. (2013). Frequency of dicentrics and contamination levels in Ukrainian children and adolescents from areas near Chernobyl 20 years after the nuclear plant accident. Int. J. Radiat. Biol..

[28] George K., Wu H., Willingham V., Cucinotta F.A. (2002). Analysis of complex-type chromosome exchanges in astronauts’ lymphocytes after space flight as a biomarker of high-LET exposure. J. Radiat. Res..

[29] TESTARD B.D., SABATIER I.L. (1997). Chromosomal aberrations induced in human lymphocytes by high-LET irradiation. Int. J. Radiat. Biol..

[30] Tanaka K., Gajendiran N., Endo S., Komatsu K., Hoshi M., Kamada N. (1999). Neutron energy-dependent initial DNA damage and chromosomal exchange. J. Radiat. Res..

[31] Bochkov N., Katosova L. (1994). Analysis of multiaberrant cells in lymphocytes of persons living in different ecological regions. Mutat. Res. Lett..

[32] Balajee A.S., Hadjidekova V. (2021). Retrospective cytogenetic analysis of unstable and stable chromosome aberrations in the victims of radiation accident in Bulgaria. Mutat. Res./Genet. Toxicol. Environ. Mutagen..

[33] Hande M.P., Azizova T.V., Burak L.E., Khokhryakov V.F., Geard C.R., Brenner D.J. (2005). Complex chromosome aberrations persist in individuals many years after occupational exposure to densely ionizing radiation: An mFISH study. Genes Chromosom. Cancer.

[34] Raap A., Tanke H. (2006). Combined binary ratio fluorescence in situ hybridiziation (COBRA-FISH): Development and applications. Cytogenet. Genome Res..

[35] Livingston G.K., Ryan T.L., Smith T.L., Escalona M.B., Foster A.E., Balajee A.S. (2019). Detection of simple, complex, and clonal chromosome translocations induced by internal radioiodine exposure: A cytogenetic follow-up case study after 25 years. Cytogenet. Genome Res..

